# Janus Kinase Inhibitors for Treatment of Palmoplantar Pustulosis, Generalized Pustular Psoriasis, and Palmoplantar Pustular Psoriasis: A Systematic Review of the Literature

**DOI:** 10.1002/hsr2.72301

**Published:** 2026-04-06

**Authors:** Mahshid Sadat Ansari, Sama Heidari, Elnaz Pourgholi, Saeed Bahramian, Nasim Tootoonchi, Seyed Mohammad Vahabi

**Affiliations:** ^1^ Department of Dermatology, Razi Hospital Tehran University of Medical Sciences Tehran Iran

**Keywords:** JAK inhibitors, JAK–STAT pathway, palmoplantar pustulosis, pustular psoriasis

## Abstract

**Introduction:**

Palmoplantar pustulosis (PPP) is a chronic, recurrent, inflammatory disease and assumed to be a subtype of psoriasis. Pustular psoriasis (PP) is a chronic inflammatory disease that is further subclassified into various entities with different presentations including generalized pustular psoriasis (GPP) and palmoplantar pustular psoriasis (PPPP). Given the central role of the JAK–STAT pathway in cytokine signaling, this systematic review evaluated the effectiveness and safety of Janus kinase inhibitors (JAK‐I) in these PP subtypes.

**Methods:**

Following PRISMA 2020 guidelines, a systematic search was conducted across PubMed/Medline, Scopus, Web of Science, and Embase up to November 13, 2025. Eligible studies included assessing JAK‐I in PPP, GPP, or PPPP. Exclusion criteria were reviews, articles without full‐text, SAPHO syndrome, and animal/in vitro studies. Risk of bias was assessed using the NHLBI quality assessment tool for clinical studies and Murad et al.'s checklist for case reports/series.

**Results:**

Thirty‐seven studies were included (29 case reports, 4 case series, and 4 clinical studies), encompassing 157 patients (60.5% female; mean age 46.8 years). Treatments involved tofacitinib, upadacitinib, baricitinib, abrocitinib, and topical ruxolitinib. In PPP, pooled meta‐analysis demonstrated a PPPASI‐50 response rate of 85.5% (95% CI, 71.3–93.3), with upadacitinib achieving 90.9% (95% CI, 81.7–95.7). Case reports and series showed 88.1% clearance or near‐clearance within a mean of 2.5 months. GPP patients (*n* = 5) achieved rapid clearance or marked improvement within 2–12 weeks. Adverse events (18.7%) were generally mild, most commonly acneiform eruptions, headache, and transient liver enzyme elevations, with no severe events reported.

**Conclusion:**

JAK‐I demonstrate high response rates and rapid improvement with manageable safety profiles. However, the current evidence is limited by small sample sizes, short follow‐up durations, and reliance on case‐based data. They represent a promising therapeutic option and warrant further evaluation in larger controlled studies to establish long‐term efficacy and safety.

## 1 Introduction

Palmoplantar pustulosis (PPP) is a chronic, recurrent, inflammatory disease characterized by sterile pustules on the palms and soles. Although lesions are primarily located on the hands and feet, they can extend to other areas, including fingers and toes [1, 2, 3]. The prevalence of PPP is higher in Japan compared to Western countries, with estimates ranging from 0.005% to 12% of the population [4, 5].

This immune‐mediated disease is assumed to be a subtype of psoriasis. Despite its localization, PPP can exhibit features similar to those of psoriasis, such as hyperkeratosis, erythema, nail involvement, psoriatic arthritis, and scaling. Moreover, lesions may develop in other body regions, and affect areas such as the upper and lower limbs [4, 6, 7, 8]. Treatment resistance is common, and is regarded as debilitating due to its negative effects on patients' functionality and overall quality of life [9, 10]. Both environmental and genetic factors are known triggers, with both innate and adaptive immune systems involved in its pathogenesis [4].

While the exact pathogenesis remains unknown, the primary site of inflammation is believed to be the acrosyringium beneath the stratum corneum. Sweat glands, typically immune‐competent structures, serve as a robust barrier protecting the skin. Furthermore, proinflammatory cytokines including interleukin‐1α (IL‐1α), interleukin‐1β (IL‐1β), and TNF‐α are usually produced in these glands. However, abnormalities and malfunctions in sweat glands' function may lead to PPP. It is posited that inflammation is initiated by IL‐1α, subsequently involving T‐cells and Langerhans cells in the skin inflammation development [4, 5, 11].

Pustular psoriasis (PP) is a chronic inflammatory disease that is further subclassified into various entities with different presentations including generalized pustular psoriasis (GPP) and palmoplantar pustular psoriasis (PPPP). GPP is characterized by widespread sterile pustules and can be associated with systemic involvement, potentially resulting in severe complications such as fever, sepsis, renal and heart failure, and respiratory distress syndrome [12]. PPPP is characterized by sterile pustules of palms and soles, accompanied by psoriatic features such as mild scaly erythema. However, distinguishing between PPP and PPPP can be challenging due to their clinical and morphological similarities. The presentation of sterile pustules on erythematous palmoplantar skin without induration and scaling is characteristic of PPP, whereas patients with psoriasis in other areas of the body exhibit typical features such as well‐demarcated, scaly plaques with pustules on the palms and soles, which is attributed to PPPP [7, 13].

Although both PPP and PP share similarities regarding the role of proinflammatory cytokines in their pathogenesis, certain cytokines, such as those associated with the IL‐36 axis, are more prominently involved in PP [10, 14, 15].

The Janus kinase and signal transducer and activator of transcription (JAK–STAT) pathway plays a critical role in cytokine signaling, and has various immune functions including T‐cells polarization, hematopoiesis, and inflammation monitoring, adipogenesis, and growth. Both cytokine receptors (types 1 and 2), as well as interferons and growth factors, are implicated in this pathway. Each heterodimeric cytokine receptor pairs with different JAKs, while homodimeric receptors specifically associate with JAK2. There are four types of JAKs consisting of JAK1, JAK2, JAK3, and TYK2 [16, 17, 18, 19, 20].

A wide range of treatment modalities is utilized for treating PPP, including phototherapy (ultraviolet) and lasers, topical therapies (corticosteroids and vitamin D derivatives), retinoids (e.g., acitretin), and biological medications such as anti‐TNF, anti‐interleukin 17, anti‐interleukin‐23, and apremilast. Nevertheless, the treatment of PP remains challenging [5].

Considering the similar treatment challenges and shared pathogenesis among pustular subtypes of psoriasis, this review aims to primarily focus on the effectiveness of Janus kinase inhibitors (JAK‐I) in treating PP, while also reviewing published studies on GPP and PPPP.

## 2 Materials and Methods

### 2.1 Search Strategy

A systematic search was conducted using related keywords/MeSH terms through PubMed/Medline, Scopus, Web of Science, and Embase until November 13, 2025 (see Supporting Information S1: File 1). It follows the 2020 guidelines of the Preferred Reporting Items for Systematic Reviews and Meta‐analyses (PRISMA) [21].

### 2.2 Eligibility Criteria and Study Selection

This systematic review included studies that investigated the effect of a JAK‐I in patients with PPP, GPP, and PPPP. Diagnostic classification of PPP and PPPP was based on the diagnosis reported by the original authors. Given the known clinical overlap between these entities and the lack of uniform diagnostic criteria across studies, histopathologic or genetic confirmation was not required. Reviews, articles without available text, studies on patients with SAPHO (synovitis, acne, pustulosis, hyperostosis, and osteitis) syndrome and animal/in vitro studies were excluded. An exception was made for one retrospective study that included SAPHO patients but reported pooled clinical outcomes for PPP, allowing inclusion of relevant data [22].

### 2.3 Data Extraction

Two independent reviewers screened the articles to exclude irrelevant ones. In case of disagreement, a third reviewer made the final decision. Data extracted included study characteristics, patient age, sex, comorbidities, disease duration, previous treatments, dosage and duration of received JAK‐I(s), other concurrent medications, outcomes, and possible adverse effects.

### 2.4 Risk of Bias Assessment

The methodological quality of the included studies was evaluated by two reviewers through consensus, using the National Heart, Lung, and Blood Institute (NHLBI) quality assessment instruments along with the checklist proposed by Murad et al. [23] for appraising case reports and case series (see Supporting Information S2: File 2).

### 2.5 Definition of Outcome

Treatment response definition was different in each study. It was considered clearance of lesions and/or change in clinical scores such as Palmoplantar Psoriasis Area and Severity Index (PPASI), Physician's Global Assessment (PGA), and Dermatology Life Quality Index (DLQI). A positive outcome reflects clearance or reduction of pustules, erythema, and scaling, and improved quality of life.

## 3 Results

**Figure 1:**
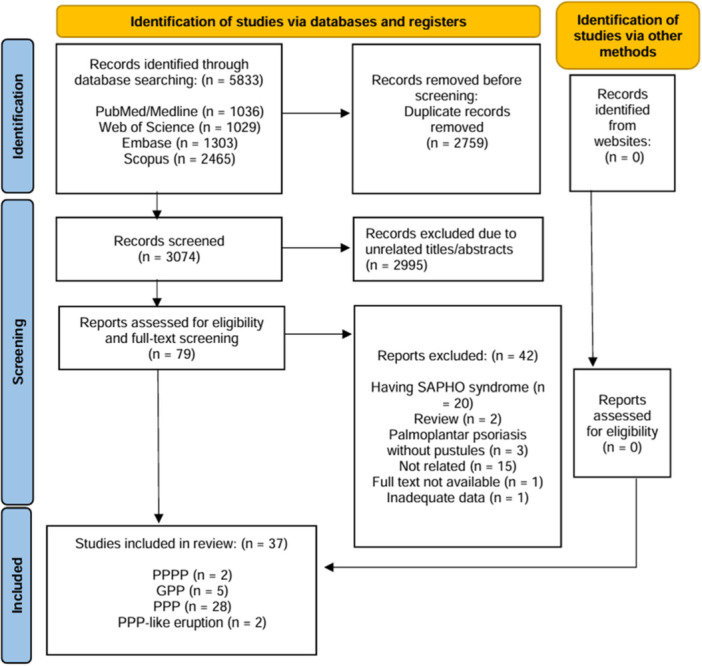
Preferred Reporting Items for Systematic Reviews and Meta‐Analyses (PRISMA). Flow chart of the number of studies identified and selected into the systematic review and meta‐analysis.

A total of 37 articles were included, comprising 29 case reports, 4 case series, and 4 clinical studies, including 3 retrospective and 1 prospective observational study (Figure 1). Of the 157 included patients, 95 (60.5%) were female. Across all 156 patients with available age data, the pooled mean age was 46.8 years, with an estimated pooled standard deviation of 13.8 years, calculated using weighted variance methods and validated imputation techniques for missing variance data. Except for two studies that reported the development of PPP‐like eruption with JAK inhibitors use, other studies showed that JAK‐I are useful in the treatment of PPPP, GPP, and PPP (Table 1). Also, one article was excluded because of the inseparable data of JAK‐I and other drugs [24].

**Table 1:** Summary of the result by each disease and each JAK inhibitor.

Disease	Number of patients	JAK inhibitor treatment	Outcome	Adverse effects
Palmoplantar pustular psoriasis	2	Tofacitinib	Clearance (100%)	None
Generalized pustular psoriasis	2	Tofacitinib	Clearance (100%)	None
	2	Upadacitinib	Clearance (100%)	None
	1	Abrocitinib	Clearance (100%)	None
Palmoplantar pustulosis	95	Upadacitinib	79 patients: PPPASI‐50: 90.9% 16 patients: clearance and almost cleared 12 (62.5%) Improvement 5 (31.3%) Remained in remission 1 (6.3%)	26 (27.4%) 11 acne 5 headache 4 elevated liver enzymes 2 dyslipidemia 2 upper respiratory infection 1 HSV reactivation 1 bronchitis and cystitis 1 hypertension
	51	Tofacitinib	29 patients: PPPASI‐50: 72.4% 22 patients: clearance and almost cleared 12 (66.67%) Improvement 6 (33.33%)	3 (5.9%) 1 dental abscess 2 gastric discomforts
	2	Baricitinib	Clearance (100%)	None
	1	Abrocitinib	Improvement (100%)	None
	1	Ruxolitinib	Clearance (100%)	None

Abbreviation: PPPASI, Palmoplantar Psoriasis Area and Severity Index.

### 3.1 Generalized Pustular Psoriasis (GPP)

Five case reports [25, 26, 27, 28, 29] of GPP were identified (three males and two females; mean age, 31.6 ± 14.21 years; range, 13–48 years) (Table 2). Treatment included tofacitinib (*n* = 2), upadacitinib (*n* = 2), and abrocitinib (*n* = 1). Four patients received JAK inhibitor monotherapy; one used glycyrrhizin, cefuroxime, and topical corticosteroids concurrently. Regarding clinical outcomes, two patients achieved complete clearance within 2 weeks, one patient reached a 60% improvement at 1 month, one patient improved from a Generalized Pustular Psoriasis Physician Global Assessment (GPPGA) score of 4 to 1 within 5 days, and another achieved near‐complete resolution by 3 months. Follow‐up (available in three cases) ranged from 3 to 4 months, with no recurrences or adverse effects reported.

**Table 2:** JAK inhibitors for the treatment of generalized pustular psoriasis.

Study	Number of patients	Comorbidities and personal history	Previous treatment	JAK inhibitor treatment	Concomitant treatment	Outcome	Follow‐up	Adverse effects
	Age‐sex							
Shreya et al. [25]	One 48‐Male	NA	CSs, ACT, CYC, MTX, ETC	Tofacitinib 5 mg twice daily	No	Clearance 2 weeks	3 months	None
Wang et al. [26]	One 21‐Male	PsA, nail involvement	ACT, MTX	Upadacitinib 15 mg daily	No	Almost cleared 3 months	3 months	None
Xiaoyuan et al. [27]	One 13‐Female	PRP, psoriasis	ACT, MTX, secukinumab	Upadacitinib 15 mg daily	No	60% resolution 1 month	NA	NA
Wang et al. [28]	One 28‐Male	NA	ACT, MTX, topical CS, an unspecified traditional Chinese medicine (TCM)	Tofacitinib 5 mg twice daily	Glycyrrhizin, cefuroxime, and hydrocortisone butyrate cream	GPPGA 4 to 1 in 5 days After 2 weeks, tofacitinib was tapered to 5 mg once daily	4 months without recurrence	None
Yang et al. [29]	One 48‐Female	NA	ACT, Prednisolone, topical CS, and tacrolimus cream	Abrocitinib 100 mg twice daily	No	Clearance 2 weeks	NA	None

Abbreviations: ACT, acitretin; CYC, cyclosporine; CSs, corticosteroids; ETC, etanercept; GPPGA, Generalized Pustular Psoriasis Physician Global Assessment; MTX, methotrexate; PRP, pityriasis rubra pilaris; PsA, psoriatic arthritis.

### 3.2 Palmoplantar Pustular Psoriasis and Palmoplantar Pustulosis

A total of 150 patients were included in this group (Tables 3 and 4); 2 with PPPP, and others had PPP.

**Table 3:** JAK inhibitors for the treatment of palmoplantar pustular psoriasis and palmoplantar pustulosis.

Study	Number of patients	Comorbidities and personal history	Previous treatment	JAK inhibitor treatment	Concomitant treatment	Outcome	Follow‐up	Adverse effects
	Age‐sex							
Juliane and Markus [30] PPPP	One 61‐Female	PsA	NA	Tofacitinib 10 mg daily	Prednisolone Tapered over 3 weeks	Clearance 4 weeks	16 months	NA
Muzumdar et al. [31] PPPP	One 45‐Female	None	Topical CSs, certolizumab, ETC, ADA, IXE, SEK, USM, GUSE, MTX	Tofacitinib 11 mg daily	No	Significant improvement 1 month	Clearance 3 months	None
Koga et al. [32] PPP	64‐Female	RA	MTX, ADA	Tofacitinib 10 mg twice daily	No	Improvement	NA	NA
Haynes et al. [33] PPP	45‐Female	Smoking, obesity, PsA, plaque psoriasis	Topical therapies (CSs, vitamin D analogs), light treatments (excimer laser, Grenz ray, PUVA) Systemic medications (MTX, ACT, CYC, ADA, USM, GUSE, SEK, APR)	Tofacitinib 5 mg twice daily	Topical CSs	Almost cleared 2 weeks	3 months	None
Mössner et al. [34] PPP	Two							
	70‐Female	PV, HTN, SH, DM, hyperthyroidism, recurrent UTI	ACT, FAE, MTX, APR, USM, brodalumab, GUSE, ADA, Anakinra, IXE	Tofacitinib 5 mg twice daily	MTX 2.5 mg/week orally, Mometasone 0.1% ointment once daily as needed	Almost cleared 12 weeks	12 weeks	None
	67‐Male	PsA, PV, UC, HTN, DM, SH, spondylosis, HTG, dementia syndrome	ACT, FAE, MTX, CYC, MMF, leflunomide, ETC, INX, ADA, APR, SEK	Tofacitinib 5 mg twice daily	Prednisolone orally 10 mg daily, then reduced and discontinue, Betamethasone 0.1% ointment once daily as needed	Almost cleared 12 weeks	12 weeks	None
Wang and Rosenbach [35] PPP	40‐Female	UC, ADA‐induced PPP	Efalizumab, CYC, MMF, PUVA, CSs, Alefacept, Isotretinoin, USM, Topical dapsone, Anakinra, MTX, ACT, APR, Tocilizumab, GUSE	Tofacitinib 5 mg twice daily	No	Clearance Some days	1 year	None
Imafuku and Maeyama [36] PPP	64‐Female	Smoking, RA, golimumab‐induced PPP	Etretinate, topical Vit D analog/CSs, with 308 nm ultraviolet therapy, APR	Baricitinib	No	Clearance 4 weeks	NA	NA
Gaiani et al. [37] PPP	Three Female							
	55‐Female	PsA, smoking	Systemic CSs, ACT, CYC, MTX, APR, USM, IXE, Brodalumab	Upadacitinib 15 mg daily	No	Clearance 12 weeks	8 months	NA
	44‐Female	Osteopenia, PV	Systemic CSs, ACT, MTX, SEK	Upadacitinib 15 mg daily	ACT	Almost cleared 4 weeks	7 months	NA
	50‐Female	Hashimoto's thyroiditis, vitiligo, uterine fibroids, depression–anxiety, fibromyalgia, plantar fasciitis	Systemic CS, CYC, SEK, MTX	Upadacitinib 15 mg daily	No	Significant improvement 4 months	5 months	NA
Gleeson et al. [38] PPP	Two							
	49‐Female	UC	Multiple standard systemic and targeted biologic therapies	Tofacitinib 10 mg twice daily then 10 mg daily	No	Clearance 1 week	14 weeks	None
	60‐Female	PsA	Multiple standard systemic and targeted biologic therapies	Tofacitinib 10 mg daily	No	Almost cleared 2 weeks	16 weeks	None
Mohr et al. [39] PPP	68‐Female	Plaque psoriasis, hypothyroidism, HChol, depression, smoking	PUVA, FAE, MTX, ACT, alitretinoin, GUSE, APR	Upadacitinib 15 mg daily	No	Almost cleared 15 weeks	NA	None
Murray et al. [40] PPP	52‐Female	RA, smoking, ADA‐induced PPP	Topical CSs	Tofacitinib 5 mg twice daily	No	Improvement 4 days	Clearance 3 months	A dental abscess
Rahbar Kooybaran et al. [41] PPP	Five							
	73‐Female	Obesity, PV, DM, HTN, SH, HChol, hyperlipidemia, hyperthyroidism, a depressive episode	FAE, MTX, APR, ACT, USM, brodalumab, GUSE, ADA, Anakinra, IXE, Tofacitinib^a^	Upadacitinib 15 mg daily	Mometasone 0.1% ointment in daily alternation with salicylvaseline 5%.	Remained in remission	20 months	Headache
	59‐Female	PsA, HTN, AA	ADA, MTX, IXE, ACT, GUSE	Upadacitinib 15 mg daily	No	Almost cleared 6 weeks	7 months	None
	60‐Female	Smoking, CSU, chronic bronchitis, dyslipidemia	MTX, dimethyl fumarate, ACT, CYC, APR	Upadacitinib 15 mg daily	No	Almost cleared 9 weeks	9 weeks	Bronchitis and cystitis
	56‐Male	Smoking, PsA, obesity, COPD, fibromyalgia, IBS	MTX, GUSE	Upadacitinib 15 mg daily	Topical vitamin D3 derivative	Almost cleared 6 weeks	6 weeks	None
	59‐Female	PsA, fibromyalgia, HTN, severe depression, effluvium	MTX, SEK, ACT	Upadacitinib 15 mg daily	Mometasone 0.1% ointment in daily	Moderate improvement 3 months	3 months	None
Wang et al. [42] PPP	Three							
	25‐Female	None	*Tripterygium wilfordii Hook F*, CYC, ACT, topical CSs	Tofacitinib 5 mg twice daily	Topical CSs, Retinol 50 mg twice daily for 4 weeks then changed to TGs 20 mg three times a day	Almost cleared 16 weeks	20 weeks	None
	55‐Male	Alopecia, nail involvement	None	Tofacitinib 5 mg twice daily	Nitrate and azole cream	Significant improvement 4 weeks	Clearance 10 weeks	None
	71‐Male	CAD, HTN, nail involvement	TGs, ACT, dapsone	Tofacitinib 5 mg twice daily	No	Clearance 16 weeks	20 weeks	None
Xu et al. [43] PPP	Six							
	45‐Male	Smoking, obesity, hyperlipidemia, urticaria	Topical therapy (CSs, retinoids, emollients) Systemic therapy (systemic retinoid, CYC)	Tofacitinib 5 mg twice daily	No	Almost cleared 4 weeks	Clearance 12 weeks	None
	58‐Male	Smoking, obesity, DM, AD	Topical therapy (CSs, Emollients) Systemic therapy (systemic retinoid)	Tofacitinib 5 mg twice daily	No	Almost cleared 4 weeks	Almost cleared 12 weeks	None
	42‐Male	Obesity	Topical therapy (CSs, vitamin D derivatives, emollients) Systemic therapy (systemic retinoid, MTX)	Tofacitinib 5 mg twice daily	No	Almost cleared 4 weeks	Clearance 12 weeks	None
	53‐Female	Obesity, DM, rhinitis, urticaria	Topical therapy (CSs, vitamin D derivatives, retinoids, keratolytic agents, emollients, phototherapy) Systemic therapy (systemic retinoid, MMF)	Tofacitinib 5 mg twice daily	No	Almost cleared 4 weeks	Almost cleared 12 weeks	None
	46‐Male	Smoking, obesity	Topical therapy (CSs, retinoids, emollients) Systemic therapy (systemic retinoid, CYC)	Tofacitinib 5 mg twice daily	No	Significant improvement 4 weeks	Almost cleared 12 weeks	None
	55‐Female	Hyperlipidemia	Topical therapy (CSs, vitamin D derivatives, emollients) Systemic therapy (systemic retinoid, MTX)	Tofacitinib 5 mg twice daily	No	Significant improvement 4 weeks	Clearance 12 weeks	None
Zhang et al. [44] PPP	One 35‐Female	SEK‐induced AA, nail involvement	calcipotriol ointment, ACT, ADA, SEK	Tofacitinib 5 mg twice daily	No	Significant improvement 3 months	Cleared 8 months	None
Zhang et al. [45] PPP	Two							
	47‐Female	PsA, thyroid nodule, anxiety, nail involvement	IXE	Upadacitinib 15 mg daily	No	Partial improvement 4 weeks	Cleared 20 weeks	None
	54‐Female	PsA, smoking, hyperthyroidism, anxiety	Topical CSs, SEK	Upadacitinib 15 mg daily	No	Partial improvement 4 weeks	Cleared 18 weeks	None
Gao et al. [46] PPP	One 47‐Male	Rhinitis, smoking, nail involvement	ACT, desloratadine, eculizumab	Abrocitinib 100 mg daily	No	Improvement 3 months	6 months	NA
Hu et al. [47] PPP	One 55‐Male	None	SEK	Upadacitinib 15 mg daily	No	Significant improvement 3 days	Almost cleared 12 weeks	Sore throat
Li et al. [48] PPP	20‐Male	AD	Topical CSs, antihistamines, traditional Chinese medicine	Baricitinib 4 mg Daily	No	Clearance 4 weeks	6 months^b^	None
Fan and Yin [49] PPP	One 40‐Male	Allergic conjunctivitis, allergic rhinitis, asthma	Topical CSs, secukinumab	Tofacitinib 5 mg twice daily	No	Clearance 3 months	6 months	No
Cramer et al. [50] PPP	One 66‐ Female	Smoker 20‐pack‐year, psoriatic arthritis	Topical CSs, phototherapy, PUVA	Ruxolitinib cream 15 mg/g twice daily	No	PPPASI 6.2 to 0.8 10 weeks	NA	No
Dong et al. [51] PPP	One 31‐Female	NA	Topical CSs, phototherapy	Tofacitinib 5 mg twice daily	No	Mostly cleared 3 months	NA stable without recurrence	No
Gu et al. [52] PPP	One 15‐Male	NA	NA	Upadacitinib 15 mg daily	No	PPPASI 18.4 to 2.6 (near clearance) 12 weeks	NA	No
Gu et al. [53] PPP	One 8‐Female	No	Calcipotriol	Upadacitinib 15 mg daily	No	PPPASI 28.8 to 2.4, NAPSI 39 to 27 (96% reduction) 8 weeks	Treatment ended after 8 weeks, and by 12 weeks, skin and nail conditions improved further	No
Yang et al. [54] PPP	One 66‐Female	NA	Topical CSs, calcipotriol, MTX, *Tripterygium wilfordii* tablets, paeoniflorin, compound glycyrrhizin, loratadine, omalizumab	Upadacitinib 15 mg daily	No	Cleared 3 months	A year with 15 mg every 5 days as a maintenance dose without recurrence	Mild acne
Hu et al. [55] TNF‐α‐induced PPP	One 27‐male	UC, HS, spondyloarthropathy	Topical CS, ACT, infliximab, ustekinumab	Upadacitinib 30 mg/day	NA	Resolution of all symptoms 4 weeks	NA	NA

Abbreviations: AA, alopecia areata; ACT, acitretin; ADA, adalimumab; AD, atopic dermatitis; APR, apremilast; CAD, coronary heart disease; COPD, chronic obstructive pulmonary disease; CSs, corticosteroids; CSU, chronic spontaneous urticaria; CYC, cyclosporine; DM, diabetes mellitus type 2; ETC, etanercept; FAE, fumaric acid esters; GUSE, guselkumab; HChol, hypercholesteremia; HS, hidradenitis suppurativa; HTG, hypertriglyceridemia; HTN, hypertension; IBS, irritable bowel syndrome; INX, infliximab; IXE, ixekizumab; MMF, mycophenolatmofetil; MTX, methotrexate; PsA, psoriatic arthritis; PUVA, psoralen plus ultraviolet‐A radiation; PV, psoriasis vulgaris; RA, rheumatoid arthritis; SEK, secukinumab; SH, steatosis hepatis; TGs, tripterygium glycosides; UC, ulcerative colitis; USM, ustekinumab; UTI, urinary tract infections; PPPASI, Palmoplantar Psoriasis Area and Severity Index.

a Had a very good response to tofacitinib in combination with MTX.

b He experienced a relapse by stopping baricitinib after 6 months which was managed again with baricitinib and resulted in clearance.

**Table 4:** Characteristics, treatment regimens, clinical outcomes, and adverse events of included clinical studies evaluating JAK inhibitors in palmoplantar pustulosis.

Study	Number of patients	Comorbidities and personal history	Previous treatment	JAK inhibitor Treatment	Concomitant treatment	Outcome	Follow‐up adverse effects (AEs)
	Age‐sex						
Zheng et al. [56] Retrospective study	20 patients 14 females 6 males mean age of 49.50 ± 10.96 years	Hyperlipidemia (4), Diabetes mellitus (3), Hypothyroidism: 2 Ulcerative colitis (1) Psoriasis: 5 AD: 3	ACT, adalimumab, ixekizumab, secukinumab, and traditional Chinese medicine	Upadacitinib 15 mg/day: 16 Upadacitinib 30 mg/day: 3 Upadacitinib 45 mg/day: 1	Topical CSs: 5 patients Ebastine: 3 patients	PPPASI: 17.69 ± 11.29 to 1.27 ± 1.41 PPPASI 50: 100% PPPASI 75: 100% PPPASI 90: 50% PPPASI 100: 20% 12 weeks	NA AE: transient increase in blood pressure (1)
Shuang et al. [57] Retrospective study	29 patients 20 Female 9 Male Median age (range): 48.2 [24–70]	Smoker: 5 Psoriasis: 5 Joint involvement: 4 Nail involvement: 7	NA	Tofacitinib	Topical CSs	PPPASI score significantly decreased from 18.62 to 6.17 PPPASI‐50: 72.41% PPP PGA ≤ 1: 62.1% 12 weeks	Mean F/U: 12.2 months: relapse: 8 Clear: 10 AEs: gastric discomfort (2)
Huang et al. [58] Prospective observational study	31 patients 15 female 16 males Mean age: 52	Smoker (26, 83.9%), hypertension (4, 12.9%), diabetes mellitus (5, 16.1%)	NA	Upadacitinib 15 mg daily	No	PPPASI 50: 28 (90.3%) PPPASI 75: 24 (77.4%) PPPASI 90: 20 (64.5%) DLQI 0/1: 27 (87.1%) complete pustule clearance: 27 (87.1%) 4 weeks	NA AEs: acne (6), headache (4), dyslipemia (2), abnormal liver enzyme (2), HSV (1), URI (1)
Du et al. [22] Retrospective study	28 patients 10 males 18 females age: 36.3 ± 10.5 years	SAPHO syndrome in eight patients smoking in two patients	ACT (15, 53.6%), TwHF (6, 21.4%), CYC (5, 17.9%), MTX (6, 21.4%), ADA (6, 21.4%), secukinumab (5, 17.9%)	Upadacitinib 15 mg daily	No	PPPASI score: 13.86 ± 2.76 to 5.56 ± 1.08 PPPASI 50:25 PPPASI 75:20 PPPASI 90: 18 12 weeks	12‐week (28 achieved disease control with no reported cases of flares) AEs: acneiform rash (4), transient transaminitis (2)

Abbreviations: ACT, acitretin; ADA, adalimumab; AD, atopic dermatitis; CSs, corticosteroids; CYC, cyclosporine; DLQI, Dermatology Life Quality Index; HSV, Herpes zoster virus; MTX, methotrexate; PPPASI, Palmoplantar Pustulosis Psoriasis Area and Severity Index; SAPHO, synovitis, acne, pustulosis, hyperostosis, osteitis; TwHF, *Tripterygium* wilfordii Hook F; URI, upper respiratory tract infection.

#### 3.2.1 Clinical Studies

Across four clinical studies [22, 56, 57, 58] comprising 108 patients with PPP, 67 were female (62.0%) (Table 4). Upadacitinib was evaluated in three studies involving 79 patients (73.1%), administered at 15 mg/day in 75 patients, 30 mg/day in 3, and 45 mg/day in 1. One study assessed tofacitinib in 29 patients (26.9%). The pooled mean age (*n* = 108) was 46.3 years.

A random‐effects meta‐analysis demonstrated that JAK inhibitors achieved a pooled PPPASI‐50 response rate of 85.5% (95% CI, 71.3–93.3; *I* ^2^ = 51%) across all four studies. When limited to upadacitinib (3 studies; 79 patients), the pooled PPPASI‐50 response rate was 90.9% (95% CI, 81.7–95.7; *I* ^2^ = 0%). Higher response thresholds showed pooled PPPASI‐75 and PPPASI‐90 rates of 78.5% (95% CI, 60.6–89.7; *I* ^2^ = 43.6%) and 60.7% (95% CI, 49.4–70.9; *I* ^2^ = 0%), respectively. In contrast, the only study evaluating tofacitinib reported a PPPASI‐50 response of 72.4% (21/29; 95% CI, 56.1%–88.7%), with insufficient data to pool PPPASI‐75 or PPPASI‐90 outcomes.

#### 3.2.2 Case Reports and Case Series

Forty‐two patients were included in this group across case reports (Table 3). Thirty‐five patients had at least one comorbidity or personal history, of which the most common ones were smoking (13), PsA (10), obesity (8), hypertension (6), and thyroid disorders (6). Also, five patients had nail involvement. Prior to intervention, 35 patients (83.3%) had received at least one systemic therapy and 10 (28.6%) received concomitant therapy with JAK inhibitors. Of these patients, 22 received tofacitinib (10–20 mg daily), 16 received upadacitinib (15 mg daily), 2 received baricitinib (4 mg daily), 1 received abrocitinib (100 mg daily), and 1 received topical ruxolitinib. Thirty‐seven (88.1%) achieved complete or almost complete clearance of lesions with a mean (±SD) treatment time of 2.5 ± 1.55 months, while the remainder showed improvement. Also, one remained in remission. The follow‐up time was available for 35 patients, with a mean (±SD) of 4.5 ± 3.96 months without any recurrences.

#### 3.2.3 Adverse Events

Across the included studies, a total of 29 adverse events were reported (29/155: 18.7%). Most events occurred in patients treated with upadacitinib (26 events; 27.4%), whereas tofacitinib accounted for 3 events (5.9%), including 1 dental abscess and 2 cases of gastric discomfort. The most frequent adverse events were acne or acneiform eruptions (11 cases), followed by headache (5 cases) and transient elevations in liver enzymes (4 cases). Less common events included dyslipidemia (2 cases) and isolated occurrences of herpes zoster, upper respiratory tract infection, bronchitis with cystitis, sore throat, and a temporary increase in blood pressure. Importantly, no severe or life‐threatening adverse events were reported, and all events resolved with conservative management or dose modification (Table 1).

### 3.3 Palmoplantar Pustulosis‐Like Eruption

Two patients [59, 60] showed PPP‐like lesions after using JAK inhibitors (Table 5). Both had an underlying rheumatologic disease, but without a history of psoriasis or any other dermatologic disease. They were managed by lowering the same JAK inhibitor dosage and adding topical corticosteroids.

**Table 5:** Palmoplantar pustulosis‐like eruption after treatment with JAK inhibitor.

Study	Previous and JAK inhibitor treatment	Effect	Follow‐up	Final plan
Age‐sex				
Disease				
Shibata et al. [59] 25‐Female JIA	Infliximab, Etanercept, Adalimumab Tofacitinib 20 mg daily	Sterile PPP developed after 10 days of initiation of tofacitinib and biopsy showed neutrophilic pustule under epidermis and leukocyte infiltration.	After discontinuing tofacitinib, the patient's pustular eruption improved, but JIA worsened. Tofacitinib was then restarted, at which point the palmoplantar pustular lesions reappeared. While a topical betamethasone ointment provided some temporary relief, the pustular lesions gradually worsened over the next 3 months of continued tofacitinib treatment.	Lowering the tofacitinib dosage to 5 mg every 2 days was adequate for joint symptoms also made the palmoplantar pustulosis‐like eruption more tolerable.
Koumaki et al. [60] 56‐Male Seropositive erosive RA	Multiple DMARDs including methotrexate and leflunomide Baricitinib 4 mg daily	After 5 years on baricitinib he developed thickened scaly skin with fissuring and pustules on both palms and soles. The histology revealed PPPP.	After discontinuing baricitinib for 2 months and using topical emollients and clobetasol, the patient's PPPP improved significantly. However, when baricitinib 4 mg daily was restarted due to worsening rheumatoid arthritis, the PPPP reappeared within 14 days, indicating baricitinib was the likely cause of the cutaneous adverse reaction.	Baricitinib dosage was lowered to 2 mg once daily, which was more tolerable for his skin condition.

Abbreviations: DMARDs, disease‐modifying antirheumatic drugs; JIA, juvenile idiopathic arthritis; RA, rheumatoid arthritis.

### 3.4 Quality Assessment

**Figure 2:**
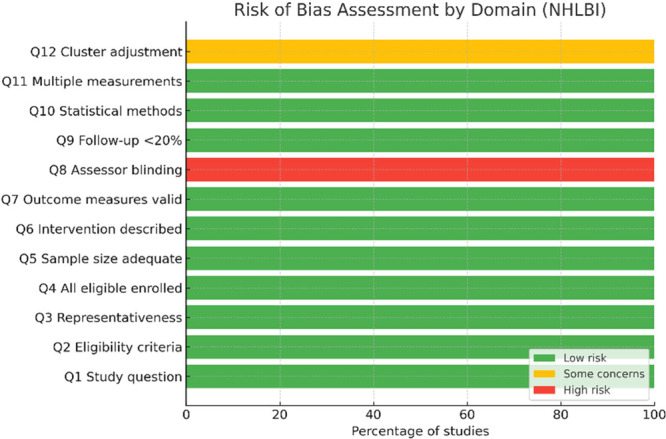
Risk of bias assessment across four clinical studies using the NHLBI quality assessment tool.

Using the NHLBI tool, all four included studies demonstrated an overall low risk of bias. Domains D2 and D4 were consistently rated as low risk across all studies, indicating clear eligibility criteria and well‐described interventions. Minor variability was observed in D1 and D3, where several items were rated as not applicable or raised some concerns, primarily due to incomplete reporting (Figure 2).

**Figure 3:**
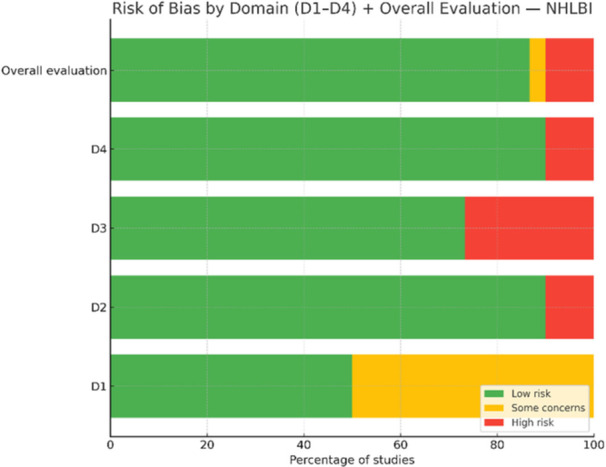
Domain‐level risk‐of‐bias assessment of the included case reports using the Murad et al. tool.

In the evaluation of case reports using the Murad and colleagues quality assessment tool, a total of 31 case reports were analyzed across four methodological domains (D1–D4) along with an overall risk‐of‐bias assessment. Of the 31 studies, 26 (83.9%) were judged to have low overall risk of bias, 1 study (3.2%) was rated as moderate risk, and 3 studies (9.7%) were rated as high risk (Figure 3).

## 4 Discussion

In the present study, we evaluate the efficacy and safety of various JAK inhibitors for treating pustular lesions, including PPP and PP. The studies we included primarily consisted of case reports and case series documenting GPP, PPPP, and PPP, with four clinical studies specifically reporting on PPP. All patients with GPP and PPPP showed a complete response to all JAK inhibitors used, including tofacitinib, upadacitinib, and abrocitinib. Regarding PPP, 90.9% of patients achieved PPPASI‐50 with upadacitinib, and 72.4% achieved PPPASI‐50 with tofacitinib in clinical studies. In case reports, 88.1% of patients demonstrated a complete or near‐complete response to treatment with tofacitinib, upadacitinib, baricitinib, abrocitinib, and topical ruxolitinib. We also reported two cases of PPP‐like eruptions, associated with the use of two different JAK inhibitors.

Apart from paradoxical reactions with JAK inhibitors, various biologics have been associated with palmoplantar pustulosis and psoriasis‐like eruptions, including anti‐TNF‐α, anti‐IL‐17, anti‐IL12/23, and anti‐IL‐6 [61, 62, 63, 64]. Although the exact pathomechanisms of these reactions remain unclear, it is postulated that an imbalance between TNF‐α inhibition and IFNα overproduction leads to an abnormal T‐cell response. Additionally, the JAK–STAT pathway may also contribute, as some interleukins are implicated when JAK inhibitors induce paradoxical eruptions [59, 60, 64].

An increased expression of IL‐17 compared to IL‐12 and IL‐23 was noted in both PPP and PPPP. IL‐17, a proinflammatory cytokine, is considered a key immunopathogenic factor in PPP and PPPP, produced by both innate (neutrophils and mast cells) and adaptive (Th17) immune system [65, 66]. Other key inflammatory factors are IL‐8, IL‐1α, IL‐1β, IL‐22, and TNF‐α [2, 8].

Previous studies have demonstrated that IL‐19 is significantly upregulated in both the blood and skin of patients with PPP, suggesting its role as a key cytokine in the disease's pathogenesis. Unlike other major cytokines, IL‐19 is primarily secreted by monocytes, activated macrophages, and dendritic cells. Moreover, IL‐19 has potential as a predictive biomarker for treatment response, as its levels have been shown to significantly decrease following successful PPP therapy [67].

Both PP and PPP are challenging diseases to treat. Various therapeutic methods have been employed, including topical treatments (e.g., steroids), phototherapy (narrowband‐ultraviolet B, psoralen and ultraviolet A), cyclosporine, methotrexate, apremilast, and biological therapies like anti‐TNF‐α and anti‐IL‐12/23 [9, 68, 69]. However, biologics have shown more promising effectiveness and better tolerability [70, 71].

Previous studies have identified anti‐TNF‐α and anti‐IL17 as the most effective biologics in treating PPP and PP, particularly at higher doses. However, increasing dosages may elevate the risk of adverse events, and the loss of responsiveness can occur during the course of therapy [70, 72].

PP variants (GPP and PPPP) as well as PPP, may share common genetic markers such as IL‐36 receptor antagonist (IL‐36RN), CARD14, AP1S3, and ATG16L1. However, no association has been identified between PPP and the primary susceptible gene of psoriasis, PSORS1, although pustular forms of psoriasis are linked to this gene [7, 10, 73]. Mutation in IL‐36RN and dysfunction in IL‐36 axis lead to the overproduction of IL‐36 in these diseases, significantly contributing to their onset [74, 75]. Despite the differences in genetic foundation, both conditions can respond to shared treatments [70].

Since understanding of the JAK–STAT pathway, the perspective on treating a range of autoimmune and autoinflammatory dermatological diseases has significantly evolved [76, 77, 78, 79]. Induction of IL‐23 through JAK–STAT signaling pathway plays a crucial role in the development of immune‐mediated diseases, by influencing the activation and proliferation of memory T‐cells responsible for IL‐17 upregulation. Studies have shown that tofacitinib which is a JAK1/3 inhibitor [32], inhibits the IL‐23/IL‐17 axis by blocking IL‐23 induced JAK–STAT signaling [80, 81]. Other JAK inhibitors exert their pharmacological effects through various pathways. Baricitinib is a JAK1/2 inhibitor that regulates both innate and adaptive immunity by modulating Th2 responses and inhibiting type‐1 IFN, IL‐12, IL‐23, and IL‐2 [82]. Upadacitinib is a selective JAK1 inhibitor that affects the activation of inflammatory T cells and reduce the inflammatory cytokines, including IL‐6, IL‐17, IL‐2, IL‐36, and IFNγ [22]. Abrocitinib is also a selective JAK1 inhibitor that reduces the proliferation of Th2 cells by inhibiting the signaling of IL‐4 and IL‐13 [83]. Since PPP, GPP, and PPPP share a common genetic and immunologic background, the mechanisms of action of each JAK inhibitor—whether through regulation or inhibition—can influence all of these diseases. This is particularly relevant as common inflammatory factors such as Th1, Th17, IL‐8, IL‐36, and interferons play key roles in the pathogenesis of the aforementioned conditions [14].

Additionally, an inhibitory approach targeting the JAK–STAT signaling pathway was reported by Hammitzsch, in which RNA‐mediated silencing of all the JAK family members (JAK1, JAK2, JAK3, and TYK2) was performed in CD4^+^ T‐cells. The study found that targeted JAK1, JAK3, and TYK2 inhibited the secretion of all IL‐17 classes (IL‐17A, IL‐17F, and IL‐22), whereas silencing of JAK2 did not significantly impact cytokine inhibition. Inhibition of STAT phosphorylation was achieved by silencing TYK2 (STAT5 inhibition), JAK2 (STAT3), and JAK1 and JAK2 (STAT1 and 3) [80, 84]. Additionally, JAK inhibitor like tofacitinib have been shown to decrease the production of other involved inflammatory cytokines such as IL‐6, IL‐8, and Interferon (IFN)γ [85, 86].

Among the common risk factors contributing to the onset of PPP, smoking and obesity are particularly influential. Smoking can increase the risk of PPP up to 32.7‐folds, with both the duration and amount of smoking being associated with disease severity. Although the exact pathoethiology remains unclear, it is believed that nicotine accumulation in sweat glands impairs neutrophil function and leads to overproduction of cytokines, such as IL‐8. Changes in the subunits of nicotinic acetylcholine receptors in keratinocytes and increased expression of interleukins like IL‐17 and IL‐36 represent additional pathological mechanisms through which smoking induces PPP [5, 87]. Obesity is considered a low‐grade inflammatory state, and as the adipose tissue accumulate, the expression of inflammatory cytokines such as TNF‐α, IL‐17, IL‐8, and IL‐6 increases [88]. In addition to diseases worsening, both risk factors can also pose challenges in the treatment course with JAK inhibitors. Other comorbidities frequently observed with PPP include hypertension, diabetes, dyslipidemia, psychological disorders, respiratory and thyroid diseases [7].

Using nonselective, pan‐JAK inhibitors has been associated with unfavorable side effects, including infections [86]. However, in our study, four out of five reported side effects were related to upadacitinib (27.4%), a selective, second‐generation JAK1 inhibitor. In more than 5% of patients treated with upadacitinib, following adverse events were commonly reported: infections (upper respiratory tract infection), nausea, diarrhea, headache, acne, and atopic dermatitis [89]. All JAK inhibitors can lead to decreased neutrophil and lymphocyte counts, a dose‐dependent incidence of herpes infection and impaired lipid profile [86, 90, 91, 92]. More serious adverse events, such as major adverse cardiovascular events, stroke, and malignancy, are less common but still possible. Therefore, considerations, such as immunizations and complete laboratory analysis, should be made prior to treatment with JAK inhibitors, especially in elderly patients [93, 94, 95].

The most prominent limitation of this study is its small sample size, which restricts meaningful comparisons of efficacy among different JAK inhibitors. Additionally, case reports constitute a substantial portion of the included literature, reducing the overall quality of the evidence. Future research on PP should prioritize controlled trials evaluating various JAK inhibitors. Also, the diagnostic classification of PPP and PPPP was based on the diagnosis reported by the original authors.

## 5 Conclusion

PP and PPP are challenging inflammatory skin diseases with a variety of treatment options, including nonbiologics and biologics. However, no definitive treatment has been established for a complete cure. In recent years, new biological therapies have shown promising results in trials, but outcomes can vary due to different factors. In some cases, biological therapies may need to be administered at higher doses, which could increase risk of adverse events. Additionally, prolonged use of biological treatments may lead to a loss of responsiveness over time, as observed in some studies. A review of articles regarding the use of JAK inhibitors in these conditions indicates a higher response rate over a shorter treatment period compared to biologics. Moreover, side effects were generally limited and manageable in most cases. However, since the studies were primarily composed of case reports and case series, future trials with JAK inhibitors are necessary to achieve more reliable comparisons.

## Author Contributions

The specific contributions of each author to this study are as follows: Conceptualization: Mahshid Sadat Ansari and Seyed Mohammad Vahabi. Data curation: Seyed Mohammad Vahabi and Saeed Bahramian. Methodology: Saeed Bahramian, Seyed Mohammad Vahabi, and Elnaz Pourgholi. Investigation: Mahshid Sadat Ansari and Nasim Tootoonchi. Supervision: Mahshid Sadat Ansari and Nasim Tootoonchi. Validation: Sama Heidari and Saeed Bahramian. Project administration: Mahshid Sadat Ansari and Seyed Mohammad Vahabi. Writing – original draft preparation: Seyed Mohammad Vahabi, Sama Heidari, Mahshid Sadat Ansari, Saeed Bahramian, and Nasim Tootoonchi. Writing – review and editing: Seyed Mohammad Vahabi, Mahshid Sadat Ansari, Sama Heidari, and Elnaz Pourgholi.

## Funding

The authors have nothing to report.

## Disclosure

The lead author Seyed Mohammad Vahabi affirms that this manuscript is an honest, accurate, and transparent account of the study being reported; that no important aspects of the study have been omitted; and that any discrepancies from the study as planned (and, if relevant, registered) have been explained.

## Ethics Statement

The authors have nothing to report.

## Conflicts of Interest

The authors declare no conflicts of interest.

## Supporting information

Supplementary File 1.

Supplementary File 2.

## Data Availability

Data sharing is not applicable to this article, as no new data were created or analyzed in this study.
