# Supplementary material for: Janus Kinase Inhibitors for Treatment of Palmoplantar Pustulosis, Generalized Pustular Psoriasis, and Palmoplantar Pustular Psoriasis: A Systematic Review of the Literature
Source: Health Sci Rep. 2026 Apr 6;9(4):e72301. doi: 10.1002/hsr2.72301 (PMC13053663; doi:10.1002/hsr2.72301)
Supplement: Supplementary file 1 [file HSR2-9-e72301-s002.docx]

**Search Query**

| **Search** | **PubMed** | **Records** |
| --- | --- | --- |
| **1** | "(("Psoriasis"[ MeSH Major Topic]) OR ("Psoriasis"[Title/Abstract]) OR ("Pustulosis of Palms and Soles"[Title/Abstract]) OR ("Pustulosis Palmaris et Plantaris"[Title/Abstract]) OR ("Palmoplantaris Pustulosis"[Title/Abstract]) OR ("Pustular Psoriasis of Palms and Soles"[Title/Abstract]) OR ("Palmoplantar pustulosis"[Title/Abstract]) OR ("Palmoplantar psoriasis"[Title/Abstract]) OR ("Generalized pustular psoriasis"[Title/Abstract]) OR ("Pustular psoriasis"[Title/Abstract])) | 67,263 |
| **2** | "Janus Kinase Inhibitors"[MeSH Terms] OR ("Janus Kinase Inhibitors"[Pharmacological Action] OR "Janus Kinase Inhibitors"[Supplementary Concept] OR "Janus Kinase Inhibitors"[All Fields] OR "Janus Kinase Inhibitors"[MeSH Terms] OR ("janus"[All Fields] AND "kinase"[All Fields] AND "inhibitors"[All Fields])) OR ("Janus Kinase Inhibitors"[Pharmacological Action] OR "Janus Kinase Inhibitors"[Supplementary Concept] OR "Janus Kinase Inhibitors"[All Fields] OR "janus kinase inhibitor"[All Fields] OR "Janus Kinase Inhibitors"[MeSH Terms] OR ("janus"[All Fields] AND "kinase"[All Fields] AND "inhibitors"[All Fields]) OR ("janus"[All Fields] AND "kinase"[All Fields] AND "inhibitor"[All Fields])) OR ("Janus Kinase Inhibitors"[Pharmacological Action] OR "Janus Kinase Inhibitors"[Supplementary Concept] OR "Janus Kinase Inhibitors"[All Fields] OR "jak inhibitor"[All Fields] OR "Janus Kinase Inhibitors"[MeSH Terms] OR ("janus"[All Fields] AND "kinase"[All Fields] AND "inhibitors"[All Fields]) OR ("jak"[All Fields] AND "inhibitor"[All Fields])) OR ("Janus Kinase Inhibitors"[Pharmacological Action] OR "Janus Kinase Inhibitors"[Supplementary Concept] OR "Janus Kinase Inhibitors"[All Fields] OR "jak inhibitors"[All Fields] OR "Janus Kinase Inhibitors"[MeSH Terms] OR ("janus"[All Fields] AND "kinase"[All Fields] AND "inhibitors"[All Fields]) OR ("jak"[All Fields] AND "inhibitors"[All Fields])) OR ("abrocitinib"[Supplementary Concept] OR "abrocitinib"[All Fields]) OR ("abrocitinib"[Supplementary Concept] OR "abrocitinib"[All Fields] OR "pf 04965842"[All Fields]) OR ("baricitinib"[Supplementary Concept] OR "baricitinib"[All Fields]) OR ("baricitinib"[Supplementary Concept] OR "baricitinib"[All Fields] OR "ly3009104"[All Fields]) OR ("baricitinib"[Supplementary Concept] OR "baricitinib"[All Fields] OR "ly 3009104"[All Fields]) OR ("baricitinib"[Supplementary Concept] OR "baricitinib"[All Fields] OR "incb028050"[All Fields]) OR ("baricitinib"[Supplementary Concept] OR "baricitinib"[All Fields] OR "incb 028050"[All Fields]) OR ("baricitinib"[Supplementary Concept] OR "baricitinib"[All Fields] OR "olumiant"[All Fields]) OR ("baricitinib"[Supplementary Concept] OR "baricitinib"[All Fields]) OR ("baricitinib"[Supplementary Concept] OR "baricitinib"[All Fields]) OR ("baricitinib"[Supplementary Concept] OR "baricitinib"[All Fields]) OR "Brepocitinib"[All Fields] OR ("4 cyclopropylamino 2 4 4 ethylsulfonyl piperazin 1 yl phenyl amino pyrimidine 5 carboxamide"[Supplementary Concept] OR "4 cyclopropylamino 2 4 4 ethylsulfonyl piperazin 1 yl phenyl amino pyrimidine 5 carboxamide"[All Fields] OR "cerdulatinib"[All Fields]) OR ("4 cyclopropylamino 2 4 4 ethylsulfonyl piperazin 1 yl phenyl amino pyrimidine 5 carboxamide"[Supplementary Concept] OR "4 cyclopropylamino 2 4 4 ethylsulfonyl piperazin 1 yl phenyl amino pyrimidine 5 carboxamide"[All Fields] OR "prt062070"[All Fields]) OR ("2 2 1h pyrrolo 2 3 b pyridin 3 yl pyrimidin 4 yl amino 2 methyl n 2 2 2 trifluoroethyl butanamide"[Supplementary Concept] OR "2 2 1h pyrrolo 2 3 b pyridin 3 yl pyrimidin 4 yl amino 2 methyl n 2 2 2 trifluoroethyl butanamide"[All Fields] OR "decernotinib"[All Fields]) OR ("2 2 1h pyrrolo 2 3 b pyridin 3 yl pyrimidin 4 yl amino 2 methyl n 2 2 2 trifluoroethyl butanamide"[Supplementary Concept] OR "2 2 1h pyrrolo 2 3 b pyridin 3 yl pyrimidin 4 yl amino 2 methyl n 2 2 2 trifluoroethyl butanamide"[All Fields] OR "vx 509"[All Fields]) OR ("delgocitinib"[Supplementary Concept] OR "delgocitinib"[All Fields]) OR ("delgocitinib"[Supplementary Concept] OR "delgocitinib"[All Fields] OR "jte 052"[All Fields]) OR ("deuruxolitinib"[Supplementary Concept] OR "deuruxolitinib"[All Fields] OR "deuruxolitinib"[All Fields]) OR ("deuruxolitinib"[Supplementary Concept] OR "deuruxolitinib"[All Fields] OR "ctp 543"[All Fields]) OR ("fedratinib"[Supplementary Concept] OR "fedratinib"[All Fields]) OR ("fedratinib"[Supplementary Concept] OR "fedratinib"[All Fields] OR "tg101348"[All Fields]) OR ("fedratinib"[Supplementary Concept] OR "fedratinib"[All Fields] OR "tg 101348"[All Fields]) OR ("fedratinib"[Supplementary Concept] OR "fedratinib"[All Fields] OR "inrebic"[All Fields]) OR ("fedratinib"[Supplementary Concept] OR "fedratinib"[All Fields] OR "fedratinib hydrochloride"[All Fields]) OR ("fedratinib"[Supplementary Concept] OR "fedratinib"[All Fields]) OR ("fedratinib"[Supplementary Concept] OR "fedratinib"[All Fields]) OR ("fedratinib"[Supplementary Concept] OR "fedratinib"[All Fields] OR "sar302503"[All Fields]) OR ("fedratinib"[Supplementary Concept] OR "fedratinib"[All Fields] OR "sar 302503"[All Fields]) OR ("fedratinib"[Supplementary Concept] OR "fedratinib"[All Fields]) OR ("fedratinib"[Supplementary Concept] OR "fedratinib"[All Fields]) OR ("glpg0634"[Supplementary Concept] OR "glpg0634"[All Fields] OR "filgotinib"[All Fields]) OR "Golidocitinib"[All Fields] OR ("itacitinib"[Supplementary Concept] OR "itacitinib"[All Fields]) OR ("ivarmacitinib"[Supplementary Concept] OR "ivarmacitinib"[All Fields]) OR ("ivarmacitinib"[Supplementary Concept] OR "ivarmacitinib"[All Fields] OR "ivarmacitinib sulfate"[All Fields]) OR ("ivarmacitinib"[Supplementary Concept] OR "ivarmacitinib"[All Fields] OR "shr0302"[All Fields]) OR ("ivarmacitinib"[Supplementary Concept] OR "ivarmacitinib"[All Fields] OR "shr0302 base"[All Fields]) OR ("lestaurtinib"[Supplementary Concept] OR "lestaurtinib"[All Fields]) OR ("lestaurtinib"[Supplementary Concept] OR "lestaurtinib"[All Fields] OR "kt 5555"[All Fields]) OR ("lestaurtinib"[Supplementary Concept] OR "lestaurtinib"[All Fields]) OR ("lestaurtinib"[Supplementary Concept] OR "lestaurtinib"[All Fields] OR "kt5555"[All Fields]) OR ("lestaurtinib"[Supplementary Concept] OR "lestaurtinib"[All Fields] OR "cep 701"[All Fields]) OR ("lestaurtinib"[Supplementary Concept] OR "lestaurtinib"[All Fields] OR "cep701"[All Fields]) OR ("lestaurtinib"[Supplementary Concept] OR "lestaurtinib"[All Fields] OR "cep 701"[All Fields]) OR ("lestaurtinib"[Supplementary Concept] OR "lestaurtinib"[All Fields]) OR ("lestaurtinib"[Supplementary Concept] OR "lestaurtinib"[All Fields]) OR ("lestaurtinib"[Supplementary Concept] OR "lestaurtinib"[All Fields]) OR "Lorpucitinib"[All Fields] OR ("n cyanomethyl 4 2 4 4 morpholinyl phenyl amino 4 pyrimidinyl benzamide"[Supplementary Concept] OR "n cyanomethyl 4 2 4 4 morpholinyl phenyl amino 4 pyrimidinyl benzamide"[All Fields] OR "momelotinib"[All Fields]) OR ("n cyanomethyl 4 2 4 4 morpholinyl phenyl amino 4 pyrimidinyl benzamide"[Supplementary Concept] OR "n cyanomethyl 4 2 4 4 morpholinyl phenyl amino 4 pyrimidinyl benzamide"[All Fields] OR "cyt 387"[All Fields]) OR ("n cyanomethyl 4 2 4 4 morpholinyl phenyl amino 4 pyrimidinyl benzamide"[Supplementary Concept] OR "n cyanomethyl 4 2 4 4 morpholinyl phenyl amino 4 pyrimidinyl benzamide"[All Fields] OR "cyt 387"[All Fields]) OR ("n cyanomethyl 4 2 4 4 morpholinyl phenyl amino 4 pyrimidinyl benzamide"[Supplementary Concept] OR "n cyanomethyl 4 2 4 4 morpholinyl phenyl amino 4 pyrimidinyl benzamide"[All Fields] OR "cyt387"[All Fields]) OR ("oclacitinib"[Supplementary Concept] OR "oclacitinib"[All Fields]) OR ("oclacitinib"[Supplementary Concept] OR "oclacitinib"[All Fields] OR "apoquel"[All Fields]) OR "Pacritinib"[All Fields] OR (("stat bull metrop insur co"[Journal] OR "nat struct biol"[Journal] OR "sb"[All Fields]) AND "1518"[All Fields]) OR "SB-1518"[All Fields] OR "SB1518"[All Fields] OR ("peficitinib"[Supplementary Concept] OR "peficitinib"[All Fields]) OR ("peficitinib"[Supplementary Concept] OR "peficitinib"[All Fields] OR "asp015k"[All Fields]) OR "Povorcitinib"[All Fields] OR ("pf 06651600"[Supplementary Concept] OR "pf 06651600"[All Fields] OR "ritlecitinib"[All Fields]) OR ("pf 06651600"[Supplementary Concept] OR "pf 06651600"[All Fields] OR "pf 06651600"[All Fields]) OR ("ruxolitinib"[Supplementary Concept] OR "ruxolitinib"[All Fields]) OR ("ruxolitinib"[Supplementary Concept] OR "ruxolitinib"[All Fields] OR "incb 018424"[All Fields]) OR ("ruxolitinib"[Supplementary Concept] OR "ruxolitinib"[All Fields] OR "incb018424"[All Fields]) OR ("ruxolitinib"[Supplementary Concept] OR "ruxolitinib"[All Fields]) OR ("ruxolitinib"[Supplementary Concept] OR "ruxolitinib"[All Fields] OR "incb 18424"[All Fields]) OR ("ruxolitinib"[Supplementary Concept] OR "ruxolitinib"[All Fields] OR "inc424"[All Fields]) OR ("ruxolitinib"[Supplementary Concept] OR "ruxolitinib"[All Fields] OR "inc 424"[All Fields]) OR ("ruxolitinib"[Supplementary Concept] OR "ruxolitinib"[All Fields] OR "ruxolitinib phosphate"[All Fields]) OR ("ruxolitinib"[Supplementary Concept] OR "ruxolitinib"[All Fields]) OR ("ruxolitinib"[Supplementary Concept] OR "ruxolitinib"[All Fields]) OR ("ruxolitinib"[Supplementary Concept] OR "ruxolitinib"[All Fields]) OR ("ruxolitinib"[Supplementary Concept] OR "ruxolitinib"[All Fields] OR "incb018424 phosphate"[All Fields]) OR ("ruxolitinib"[Supplementary Concept] OR "ruxolitinib"[All Fields]) OR ("ruxolitinib"[Supplementary Concept] OR "ruxolitinib"[All Fields] OR "jakafi"[All Fields]) OR ("ruxolitinib"[Supplementary Concept] OR "ruxolitinib"[All Fields] OR "jakavi"[All Fields]) OR ("ruxolitinib"[Supplementary Concept] OR "ruxolitinib"[All Fields] OR "opzelura"[All Fields]) OR ("tofacitinib"[Supplementary Concept] OR "tofacitinib"[All Fields] OR "tofacitinib s"[All Fields]) OR ("tofacitinib"[Supplementary Concept] OR "tofacitinib"[All Fields] OR "tasocitinib"[All Fields]) OR ("tofacitinib"[Supplementary Concept] OR "tofacitinib"[All Fields] OR "cp 690 550"[All Fields]) OR ("tofacitinib"[Supplementary Concept] OR "tofacitinib"[All Fields] OR "cp 690550"[All Fields]) OR ("tofacitinib"[Supplementary Concept] OR "tofacitinib"[All Fields] OR "cp 690 550"[All Fields]) OR ("tofacitinib"[Supplementary Concept] OR "tofacitinib"[All Fields] OR "cp 690550"[All Fields]) OR ("tofacitinib"[Supplementary Concept] OR "tofacitinib"[All Fields] OR "cp690550"[All Fields]) OR ("tofacitinib"[Supplementary Concept] OR "tofacitinib"[All Fields] OR "tofacitinib citrate"[All Fields]) OR ("tofacitinib"[Supplementary Concept] OR "tofacitinib"[All Fields] OR "xeljanz"[All Fields] OR "tofacitinib s"[All Fields]) OR ("upadacitinib"[Supplementary Concept] OR "upadacitinib"[All Fields]) OR ("upadacitinib"[Supplementary Concept] OR "upadacitinib"[All Fields] OR "abt 494"[All Fields]) OR ("upadacitinib"[Supplementary Concept] OR "upadacitinib"[All Fields] OR "rinvoq"[All Fields]) | 23,581 |
| **Final Search** | #1 AND #2 | 1,036 |

| **Search** | **Scopus** | **Records** |
| --- | --- | --- |
| **1** | ((TITLE-ABS-KEY("Psoriasis")) OR (TITLE-ABS-KEY("Pustulosis of Palms and Soles")) OR (TITLE-ABS-KEY("Pustulosis Palmaris et Plantaris")) OR (TITLE-ABS-KEY("Palmoplantaris Pustulosis")) OR (TITLE-ABS-KEY("Pustular Psoriasis of Palms and Soles")) OR (TITLE-ABS-KEY("Palmoplantar pustulosis")) OR (TITLE-ABS-KEY("Palmoplantar psoriasis")) OR (TITLE-ABS-KEY("Generalized pustular psoriasis")) OR (TITLE-ABS-KEY("Pustular psoriasis"))) | 102,960 |
| **2** | (TITLE-ABS-KEY("Janus Kinase Inhibitors") OR TITLE-ABS-KEY("Janus Kinase Inhibitor") OR TITLE-ABS-KEY("JAK Inhibitor") OR TITLE-ABS-KEY("JAK Inhibitors") OR TITLE-ABS-KEY("Abrocitinib") OR TITLE-ABS-KEY("PF-04965842") OR TITLE-ABS-KEY("Baricitinib") OR TITLE-ABS-KEY("LY3009104") OR TITLE-ABS-KEY("LY-3009104") OR TITLE-ABS-KEY("INCB028050") OR TITLE-ABS-KEY("INCB-028050") OR TITLE-ABS-KEY("Olumiant") OR TITLE-ABS-KEY("baricitinib phosphate") OR TITLE-ABS-KEY("baricitinib phosphate salt") OR TITLE-ABS-KEY("INCB-28050") OR TITLE-ABS-KEY("Brepocitinib") OR TITLE-ABS-KEY("Cerdulatinib") OR TITLE-ABS-KEY("PRT062070") OR TITLE-ABS-KEY("Decernotinib") OR TITLE-ABS-KEY("VX-509") OR TITLE-ABS-KEY("delgocitinib") OR TITLE-ABS-KEY("JTE-052") OR TITLE-ABS-KEY("Deuruxolitinib") OR TITLE-ABS-KEY("CTP-543") OR TITLE-ABS-KEY("Fedratinib") OR TITLE-ABS-KEY("TG101348") OR TITLE-ABS-KEY("TG-101348") OR TITLE-ABS-KEY("Inrebic") OR TITLE-ABS-KEY("fedratinib hydrochloride") OR TITLE-ABS-KEY("fedratinib dihydrochloride monohydrate") OR TITLE-ABS-KEY("fedratinib hydrochloride monohydrate") OR TITLE-ABS-KEY("SAR302503") OR TITLE-ABS-KEY("SAR-302503") OR TITLE-ABS-KEY("SAR-302503A") OR TITLE-ABS-KEY("SAR302503A") OR TITLE-ABS-KEY("Filgotinib") OR TITLE-ABS-KEY("Golidocitinib") OR TITLE-ABS-KEY("Itacitinib") OR TITLE-ABS-KEY("Ivarmacitinib") OR TITLE-ABS-KEY("ivarmacitinib sulfate") OR TITLE-ABS-KEY("SHR0302") OR TITLE-ABS-KEY("SHR0302 base") OR TITLE-ABS-KEY("Lestaurtinib") OR TITLE-ABS-KEY("KT-5555") OR TITLE-ABS-KEY("KT-555") OR TITLE-ABS-KEY("KT5555") OR TITLE-ABS-KEY("CEP-701") OR TITLE-ABS-KEY("CEP701") OR TITLE-ABS-KEY("CEP 701") OR TITLE-ABS-KEY("SP-924") OR TITLE-ABS-KEY("SP924") OR TITLE-ABS-KEY("SPM-924") OR TITLE-ABS-KEY("Lorpucitinib") OR TITLE-ABS-KEY("Momelotinib") OR TITLE-ABS-KEY("CYT 387") OR TITLE-ABS-KEY("CYT-387") OR TITLE-ABS-KEY("CYT387") OR TITLE-ABS-KEY("Oclacitinib") OR TITLE-ABS-KEY("apoquel") OR TITLE-ABS-KEY("Orolitinib") OR TITLE-ABS-KEY("Pacritinib") OR TITLE-ABS-KEY("SB 1518") OR TITLE-ABS-KEY("SB-1518") OR TITLE-ABS-KEY("SB1518") OR TITLE-ABS-KEY("Peficitinib") OR TITLE-ABS-KEY("ASP015K") OR TITLE-ABS-KEY("Povorcitinib") OR TITLE-ABS-KEY("Ritlecitinib") OR TITLE-ABS-KEY("PF-06651600") OR TITLE-ABS-KEY("Ruxolitinib") OR TITLE-ABS-KEY("INCB-018424") OR TITLE-ABS-KEY("INCB018424") OR TITLE-ABS-KEY("INCA24") OR TITLE-ABS-KEY("INCB-18424") OR TITLE-ABS-KEY("INC424") OR TITLE-ABS-KEY("INC-424") OR TITLE-ABS-KEY("ruxolitinib phosphate") OR TITLE-ABS-KEY("ruxolitinib monophosphate") OR TITLE-ABS-KEY("INCB-18424 phosphate") OR TITLE-ABS-KEY("INCB-018424 phosphate") OR TITLE-ABS-KEY("INCB018424 phosphate") OR TITLE-ABS-KEY("INCB-018424 salt") OR TITLE-ABS-KEY("Jakafi") OR TITLE-ABS-KEY("Jakavi") OR TITLE-ABS-KEY("opzelura") OR TITLE-ABS-KEY("Tofacitinib") OR TITLE-ABS-KEY("tasocitinib") OR TITLE-ABS-KEY("CP 690,550") OR TITLE-ABS-KEY("CP 690550") OR TITLE-ABS-KEY("CP-690,550") OR TITLE-ABS-KEY("CP-690550") OR TITLE-ABS-KEY("CP690550") OR TITLE-ABS-KEY("tofacitinib citrate") OR TITLE-ABS-KEY("Xeljanz") OR TITLE-ABS-KEY("Upadacitinib") OR TITLE-ABS-KEY("ABT-494") OR TITLE-ABS-KEY("Rinvoq")) | 32,262 |
| **Final Search** | #1 AND #2 | 2,465 |

| **Search** | **Web of Science** | **Records** |
| --- | --- | --- |
| **1** | ((TS="Psoriasis") OR (TS="Pustulosis of Palms and Soles") OR (TS="Pustulosis Palmaris et Plantaris") OR (TS="Palmoplantaris Pustulosis") OR (TS="Pustular Psoriasis of Palms and Soles") OR (TS="Palmoplantar pustulosis") OR (TS="Palmoplantar psoriasis") OR (TS="Generalized pustular psoriasis") OR (TS="Pustular psoriasis")) | 62,479 |
| **2** | (TS="Janus Kinase Inhibitors" OR TS="Janus Kinase Inhibitor" OR TS="JAK Inhibitor" OR TS="JAK Inhibitors" OR TS="Abrocitinib" OR TS="PF-04965842" OR TS="Baricitinib" OR TS="LY3009104" OR TS="LY-3009104" OR TS="INCB028050" OR TS="INCB-028050" OR TS="Olumiant" OR TS="baricitinib phosphate" OR TS="baricitinib phosphate salt" OR TS="INCB-28050" OR TS="Brepocitinib" OR TS="Cerdulatinib" OR TS="PRT062070" OR TS="Decernotinib" OR TS="VX-509" OR TS="delgocitinib" OR TS="JTE-052" OR TS="Deuruxolitinib" OR TS="CTP-543" OR TS="Fedratinib" OR TS="TG101348" OR TS="TG-101348" OR TS="Inrebic" OR TS="fedratinib hydrochloride" OR TS="fedratinib dihydrochloride monohydrate" OR TS="fedratinib hydrochloride monohydrate" OR TS="SAR302503" OR TS="SAR-302503" OR TS="SAR-302503A" OR TS="SAR302503A" OR TS="Filgotinib" OR TS="Golidocitinib" OR TS="Itacitinib" OR TS="Ivarmacitinib" OR TS="ivarmacitinib sulfate" OR TS="SHR0302" OR TS="SHR0302 base" OR TS="Lestaurtinib" OR TS="KT-5555" OR TS="KT-555" OR TS="KT5555" OR TS="CEP-701" OR TS="CEP701" OR TS="CEP 701" OR TS="SP-924" OR TS="SP924" OR TS="SPM-924" OR TS="Lorpucitinib" OR TS="Momelotinib" OR TS="CYT 387" OR TS="CYT-387" OR TS="CYT387" OR TS="Oclacitinib" OR TS="apoquel" OR TS="Orolitinib" OR TS="Pacritinib" OR TS="SB 1518" OR TS="SB-1518" OR TS="SB1518" OR TS="Peficitinib" OR TS="ASP015K" OR TS="Povorcitinib" OR TS="Ritlecitinib" OR TS="PF-06651600" OR TS="Ruxolitinib" OR TS="INCB-018424" OR TS="INCB018424" OR TS="INCA24" OR TS="INCB-18424" OR TS="INC424" OR TS="INC-424" OR TS="ruxolitinib phosphate" OR TS="ruxolitinib monophosphate" OR TS="INCB-18424 phosphate" OR TS="INCB-018424 phosphate" OR TS="INCB018424 phosphate" OR TS="INCB-018424 salt" OR TS="Jakafi" OR TS="Jakavi" OR TS="opzelura" OR TS="Tofacitinib" OR TS="tasocitinib" OR TS="CP 690,550" OR TS="CP 690550" OR TS="CP-690,550" OR TS="CP-690550" OR TS="CP690550" OR TS="tofacitinib citrate" OR TS="Xeljanz" OR TS="Upadacitinib" OR TS="ABT-494" OR TS="Rinvoq") | 22,296 |
| **Final Search** | #1 AND #2 | 1,029 |

| **Search** | **Embase** | **Records** |
| --- | --- | --- |
| **1** | (('Psoriasis':ab,ti) OR ('Pustulosis of Palms and Soles':ab,ti) OR ('Pustulosis Palmaris et Plantaris':ab,ti) OR ('Palmoplantaris Pustulosis':ab,ti) OR ('Pustular Psoriasis of Palms and Soles':ab,ti) OR ('Palmoplantar pustulosis':ab,ti) OR ('Palmoplantar psoriasis':ab,ti) OR ('Generalized pustular psoriasis':ab,ti) OR ('Pustular psoriasis':ab,ti)) | 92,397 |
| **2** | ('Janus Kinase Inhibitors':ab,ti OR 'Janus Kinase Inhibitor':ab,ti OR 'JAK Inhibitor':ab,ti OR 'JAK Inhibitors':ab,ti OR 'Abrocitinib':ab,ti OR 'PF-04965842':ab,ti OR 'Baricitinib':ab,ti OR 'LY3009104':ab,ti OR 'LY-3009104':ab,ti OR 'INCB028050':ab,ti OR 'INCB-028050':ab,ti OR 'Olumiant':ab,ti OR 'baricitinib phosphate':ab,ti OR 'baricitinib phosphate salt':ab,ti OR 'INCB-28050':ab,ti OR 'Brepocitinib':ab,ti OR 'Cerdulatinib':ab,ti OR 'PRT062070':ab,ti OR 'Decernotinib':ab,ti OR 'VX-509':ab,ti OR 'delgocitinib':ab,ti OR 'JTE-052':ab,ti OR 'Deuruxolitinib':ab,ti OR 'CTP-543':ab,ti OR 'Fedratinib':ab,ti OR 'TG101348':ab,ti OR 'TG-101348':ab,ti OR 'Inrebic':ab,ti OR 'fedratinib hydrochloride':ab,ti OR 'fedratinib dihydrochloride monohydrate':ab,ti OR 'fedratinib hydrochloride monohydrate':ab,ti OR 'SAR302503':ab,ti OR 'SAR-302503':ab,ti OR 'SAR-302503A':ab,ti OR 'SAR302503A':ab,ti OR 'Filgotinib':ab,ti OR 'Golidocitinib':ab,ti OR 'Itacitinib':ab,ti OR 'Ivarmacitinib':ab,ti OR 'ivarmacitinib sulfate':ab,ti OR 'SHR0302':ab,ti OR 'SHR0302 base':ab,ti OR 'Lestaurtinib':ab,ti OR 'KT-5555':ab,ti OR 'KT-555':ab,ti OR 'KT5555':ab,ti OR 'CEP-701':ab,ti OR 'CEP701':ab,ti OR 'CEP 701':ab,ti OR 'SP-924':ab,ti OR 'SP924':ab,ti OR 'SPM-924':ab,ti OR 'Lorpucitinib':ab,ti OR 'Momelotinib':ab,ti OR 'CYT 387':ab,ti OR 'CYT-387':ab,ti OR 'CYT387':ab,ti OR 'Oclacitinib':ab,ti OR 'apoquel':ab,ti OR 'Orolitinib':ab,ti OR 'Pacritinib':ab,ti OR 'SB 1518':ab,ti OR 'SB-1518':ab,ti OR 'SB1518':ab,ti OR 'Peficitinib':ab,ti OR 'ASP015K':ab,ti OR 'Povorcitinib':ab,ti OR 'Ritlecitinib':ab,ti OR 'PF-06651600':ab,ti OR 'Ruxolitinib':ab,ti OR 'INCB-018424':ab,ti OR 'INCB018424':ab,ti OR 'INCA24':ab,ti OR 'INCB-18424':ab,ti OR 'INC424':ab,ti OR 'INC-424':ab,ti OR 'ruxolitinib phosphate':ab,ti OR 'ruxolitinib monophosphate':ab,ti OR 'INCB-18424 phosphate':ab,ti OR 'INCB-018424 phosphate':ab,ti OR 'INCB018424 phosphate':ab,ti OR 'INCB-018424 salt':ab,ti OR 'Jakafi':ab,ti OR 'Jakavi':ab,ti OR 'opzelura':ab,ti OR 'Tofacitinib':ab,ti OR 'tasocitinib':ab,ti OR 'CP 690,550':ab,ti OR 'CP 690550':ab,ti OR 'CP-690,550':ab,ti OR 'CP-690550':ab,ti OR 'CP690550':ab,ti OR 'tofacitinib citrate':ab,ti OR 'Xeljanz':ab,ti OR 'Upadacitinib':ab,ti OR 'ABT-494':ab,ti OR 'Rinvoq':ab,ti) | 30,087 |
| **Final Search** | #1 AND #2 | 1,303 |

Search Date: November 13, 2025

Total records: 5833

Duplicate: 1819

Remained: 4014
