# Supplementary material for: Janus Kinase Inhibitors for Treatment of Palmoplantar Pustulosis, Generalized Pustular Psoriasis, and Palmoplantar Pustular Psoriasis: A Systematic Review of the Literature
Source: Health Sci Rep. 2026 Apr 6;9(4):e72301. doi: 10.1002/hsr2.72301 (PMC13053663; doi:10.1002/hsr2.72301)
Supplement: Supplementary file 2 [file HSR2-9-e72301-s001.docx]

|  | D1 | D2 | | D3 | | | | D4 | Overall |
| --- | --- | --- | --- | --- | --- | --- | --- | --- | --- |
|  | Q1 | Q2 | Q3 | Q4 | Q5 | Q6 | Q7 | Q8 |  |
| Shreya et. al. | NA | Yes | Yes | Yes | Yes | No | Yes | Yes | Low |
| Wang X. et. al | NA | Yes | Yes | Yes | Yes | No | Yes | Yes | Low |
| Muzumdar et. al | Yes | Yes | Yes | Yes | Yes | No | Yes | Yes | Low |
| Juliane et. al | NA | Yes | Yes | No | Yes | No | Yes | Yes | Low |
| Koga et. al | NA | Yes | Yes | Yes | Yes | No | No | Yes | Low |
| Haynes et. al | NA | Yes | Yes | No | Yes | No | Yes | Yes | Low |
| Mössner et. al | Yes | Yes | Yes | No | Yes | No | Yes | Yes | Low |
| Wang YA. et. al | NA | Yes | Yes | Yes | Yes | No | Yes | Yes | Low |
| Imafuku et. al | NA | Yes | Yes | Yes | Yes | No | No | Yes | Low |
| Gaiani et. al | Yes | Yes | Yes | Yes | Yes | No | Yes | Yes | Low |
| Gleeson et. al | Yes | Yes | Yes | Yes | Yes | No | Yes | Yes | Low |
| Mohr et. al | NA | Yes | Yes | Yes | Yes | No | Yes | Yes | Low |
| Murray et. al | NA | Yes | Yes | Yes | Yes | No | Yes | Yes | Low |
| Rahbar Kooybaran et. al | Yes | Yes | Yes | Yes | Yes | No | Yes | Yes | Low |
| Wang T. et. al | Yes | Yes | Yes | No | Yes | No | Yes | Yes | Low |
| Xu et. al | Yes | Yes | Yes | Yes | Yes | No | Yes | Yes | Low |
| Zhang C. et. al | NA | Yes | Yes | Yes | Yes | No | Yes | Yes | Low |
| Zhang Z. et. al | Yes | Yes | Yes | Yes | Yes | No | Yes | Yes | Low |
| Gao et. al | NA | Yes | Yes | Yes | Yes | No | Yes | Yes | Low |
| Hu et. al | Yes | Yes | Yes | Yes | Yes | No | Yes | Yes | Low |
| Li et. al | NA | Yes | Yes | Yes | Yes | No | Yes | Yes | Low |
| Shibata et. al | NA | Yes | Yes | Yes | Yes | Yes | No | Yes | Low |
| Koumaki et. al | NA | No | Yes | No | Yes | Yes | Yes | Yes | Moderate |
| Fan et. al | Yes | Yes | No | No | Yes | Yes | Yes | No | High |
| Cramer et. al | Yes | Yes | Yes | Yes | Yes | No | Yes | Yes | Low |
| Gu2 et al. | Yes | Yes | Yes | Yes | Yes | No | Yes | Yes | Low |
| Gu et. al | Yes | Yes | Yes | Yes | Yes | No | Yes | Yes | Low |
| Yang et. al | Yes | Yes | Yes | Yes | Yes | No | Yes | Yes | Low |
| Hu et. al | Yes | Yes | No | Yes | Yes | No | No | No | High |
| Dong et. al | Yes | Yes | No | No | Yes | No | No | No | High |

**Leading explanatory questions**

1. Does the patient(s) represent(s) the whole experience of the investigator (centre) or is the selection method unclear to the extent that other patients with similar presentation may not have been reported?

2. Was the exposure adequately ascertained?

3. Was the outcome adequately ascertained?

4. Were other alternative causes that may explain the observation ruled out?

5. Was there a challenge/rechallenge phenomenon?

6. Was there a dose–response effect?

7. Was follow-up long enough for outcomes to occur?

8. Is the case(s) described with sufficient details to allow other investigators to replicate the research or to allow practitioners make inferences related to their own practice?

| **criteria** | **Yes** | **No** | **Other (CD, NR, NA)*** |
| --- | --- | --- | --- |
| 1. Was the study question or objective clearly stated? | * |  |  |
| 2. Were eligibility/selection criteria for the study population prespecified and clearly described? | * |  |  |
| 3. Were the participants in the study representative of those who would be eligible for the test/service/intervention in the general or clinical population of interest? | * |  |  |
| 4. Were all eligible participants that met the prespecified entry criteria enrolled? | * |  |  |
| 5. Was the sample size sufficiently large to provide confidence in the findings? | * |  |  |
| 6. Was the test/service/intervention clearly described and delivered consistently across the study population? | * |  |  |
| 7. Were the outcome measures prespecified, clearly defined, valid, reliable, and assessed consistently across all study participants? | * |  |  |
| 8. Were the people assessing the outcomes blinded to the participants' exposures/interventions? |  | * |  |
| 9. Was the loss to follow-up after baseline 20% or less? Were those lost to follow-up accounted for in the analysis? | * |  |  |
| 10. Did the statistical methods examine changes in outcome measures from before to after the intervention? Were statistical tests done that provided p values for the pre-to-post changes? | * |  |  |
| 11. Were outcome measures of interest taken multiple times before the intervention and multiple times after the intervention (i.e., did they use an interrupted time-series design)? | * |  |  |
| 12. If the intervention was conducted at a group level (e.g., a whole hospital, a community, etc.) did the statistical analysis take into account the use of individual-level data to determine effects at the group level? |  |  | * |

Lyu 2025: global assessment: low risk of bias

| **criteria** | **Yes** | **No** | **Other (CD, NR, NA)*** |
| --- | --- | --- | --- |
| 1. Was the study question or objective clearly stated? | * |  |  |
| 2. Were eligibility/selection criteria for the study population prespecified and clearly described? | * |  |  |
| 3. Were the participants in the study representative of those who would be eligible for the test/service/intervention in the general or clinical population of interest? | * |  |  |
| 4. Were all eligible participants that met the prespecified entry criteria enrolled? | * |  |  |
| 5. Was the sample size sufficiently large to provide confidence in the findings? | * |  |  |
| 6. Was the test/service/intervention clearly described and delivered consistently across the study population? | * |  |  |
| 7. Were the outcome measures prespecified, clearly defined, valid, reliable, and assessed consistently across all study participants? | * |  |  |
| 8. Were the people assessing the outcomes blinded to the participants' exposures/interventions? |  | * |  |
| 9. Was the loss to follow-up after baseline 20% or less? Were those lost to follow-up accounted for in the analysis? | * |  |  |
| 10. Did the statistical methods examine changes in outcome measures from before to after the intervention? Were statistical tests done that provided p values for the pre-to-post changes? | * |  |  |
| 11. Were outcome measures of interest taken multiple times before the intervention and multiple times after the intervention (i.e., did they use an interrupted time-series design)? | * |  |  |
| 12. If the intervention was conducted at a group level (e.g., a whole hospital, a community, etc.) did the statistical analysis take into account the use of individual-level data to determine effects at the group level? |  |  | * |

Zheng 2025: low risk of bias

| **criteria** | **Yes** | **No** | **Other (CD, NR, NA)*** |
| --- | --- | --- | --- |
| 1. Was the study question or objective clearly stated? | * |  |  |
| 2. Were eligibility/selection criteria for the study population prespecified and clearly described? | * |  |  |
| 3. Were the participants in the study representative of those who would be eligible for the test/service/intervention in the general or clinical population of interest? | * |  |  |
| 4. Were all eligible participants that met the prespecified entry criteria enrolled? | * |  |  |
| 5. Was the sample size sufficiently large to provide confidence in the findings? | * |  |  |
| 6. Was the test/service/intervention clearly described and delivered consistently across the study population? | * |  |  |
| 7. Were the outcome measures prespecified, clearly defined, valid, reliable, and assessed consistently across all study participants? | * |  |  |
| 8. Were the people assessing the outcomes blinded to the participants' exposures/interventions? |  | * |  |
| 9. Was the loss to follow-up after baseline 20% or less? Were those lost to follow-up accounted for in the analysis? | * |  |  |
| 10. Did the statistical methods examine changes in outcome measures from before to after the intervention? Were statistical tests done that provided p values for the pre-to-post changes? | * |  |  |
| 11. Were outcome measures of interest taken multiple times before the intervention and multiple times after the intervention (i.e., did they use an interrupted time-series design)? | * |  |  |
| 12. If the intervention was conducted at a group level (e.g., a whole hospital, a community, etc.) did the statistical analysis take into account the use of individual-level data to determine effects at the group level? |  |  | * |

Huang 2025: low risk of bias

| **criteria** | **Yes** | **No** | **Other (CD, NR, NA)*** |
| --- | --- | --- | --- |
| 1. Was the study question or objective clearly stated? | * |  |  |
| 2. Were eligibility/selection criteria for the study population prespecified and clearly described? | * |  |  |
| 3. Were the participants in the study representative of those who would be eligible for the test/service/intervention in the general or clinical population of interest? | * |  |  |
| 4. Were all eligible participants that met the prespecified entry criteria enrolled? | * |  |  |
| 5. Was the sample size sufficiently large to provide confidence in the findings? | * |  |  |
| 6. Was the test/service/intervention clearly described and delivered consistently across the study population? | * |  |  |
| 7. Were the outcome measures prespecified, clearly defined, valid, reliable, and assessed consistently across all study participants? | * |  |  |
| 8. Were the people assessing the outcomes blinded to the participants' exposures/interventions? |  | * |  |
| 9. Was the loss to follow-up after baseline 20% or less? Were those lost to follow-up accounted for in the analysis? | * |  |  |
| 10. Did the statistical methods examine changes in outcome measures from before to after the intervention? Were statistical tests done that provided p values for the pre-to-post changes? | * |  |  |
| 11. Were outcome measures of interest taken multiple times before the intervention and multiple times after the intervention (i.e., did they use an interrupted time-series design)? | * |  |  |
| 12. If the intervention was conducted at a group level (e.g., a whole hospital, a community, etc.) did the statistical analysis take into account the use of individual-level data to determine effects at the group level? |  |  | * |

Du 2025: low risk of bias
